# ^89^Zr-cetuximab PET imaging in patients with advanced colorectal cancer

**DOI:** 10.18632/oncotarget.4672

**Published:** 2015-07-23

**Authors:** Catharina Willemien Menke-van der Houven van Oordt, Elske C. Gootjes, Marc C. Huisman, Danielle J. Vugts, Chantal Roth, Anne Marije Luik, Emma R. Mulder, Robert C. Schuit, Ronald Boellaard, Otto S. Hoekstra, Guus AMS van Dongen, Henk M.W. Verheul

**Affiliations:** ^1^ Dept of Medical Oncology, VU University Medical Center, Amsterdam, The Netherlands; ^2^ Dept of Radiology and Nuclear Medicine, VU University Medical Center, Amsterdam, The Netherlands

**Keywords:** immunoPET, cetuximab, colorectal cancer, ^89^zirconium, treatment selection

## Abstract

Monoclonal antibodies (mAbs) against the epidermal growth factor receptor (EGFR) are used in the treatment of advanced colorectal cancer (mCRC). Approximately 50% of patients benefit despite patient selection for RAS wild type (wt) tumors. Based on the hypothesis that tumor targeting is required for clinical benefit of anti-EGFR treatment, biodistribution and tumor uptake of ^89^Zr-cetuximab by Positron Emission Tomography (PET), combining the sensitivity of PET with the specificity of cetuximab for EGFR was evaluated. Ten patients with wt K-RAS mCRC received 37 ± 1 MBq ^89^Zr-cetuximab directly (<2 h) after the first therapeutic dose of cetuximab. PET-scans were performed from 1 hour to 10 days post injection (p.i.). Biodistribution was determined for blood and organs. Uptake in tumor lesions was quantified by Standardized Uptake Value (SUV) and related to response. In 6 of 10 patients ^89^Zr-cetuximab uptake in tumor lesions was detected. Four of 6 patients with ^89^Zr-cetuximab uptake had clinical benefit, while progressive disease was observed in 3 of 4 patients without ^89^Zr-cetuximab uptake. Taken together, tumor uptake of ^89^Zr-cetuximab can be visualized by PET imaging. The strong relation between uptake and response warrants further clinical validation as an innovative selection method for cetuximab treatment in patients with wt RAS mCRC.

## INTRODUCTION

Systemic treatment for patients with RAS wild type (wt) colorectal cancer (mCRC) includes anti– epidermal growth factor receptor (EGFR) monoclonal antibody (mAb) treatment with either cetuximab or panitumumab as monotherapy or in combination with chemotherapy [1]. Binding of anti-EGFR mAb prevents ligand binding to its receptor, induces receptor internalization and causes inhibition of the receptor tyrosine kinase activity, thereby interfering with cell growth, differentiation, proliferation, apoptosis and cellular invasiveness [2]. Selection of patients who will benefit from this therapy remains an area of ongoing research. Patients with mCRC harboring a K-RAS mutation [3–5] or N-RAS mutation [6] do not respond to anti-EGFR treatment. However, despite selection based on mutational status, clinical benefit (complete or partial resonse and stable disease according to RECIST 1.1) to single agent cetuximab is observed in approximately half of the patients with wt RAS mCRC [6]. Additional mutations (such as BRAF) may play a role, but have not proven to be definitive biomarkers for response [7]. Variability in pharmacokinetics of the antibody may also play a role in its clinical efficacy. It can be influenced by the expression level of the antigen throughout the body in addition to the expression level in tumor lesions. EGFR is highly expressed on hepatocytes, possibly leading to sequestration of anti-EGFR mAbs in normal liver tissue. This may result in insufficient circulating anti-EGFR mAbs to reach tumor lesions, prohibiting antitumor activity. Cetuximab through levels correlate with progression free survival, supporting the hypothesis that cetuximab availability is crucial for its antitumor activity [8]. The observation that increased skin toxicity is associated with a favorable response might also be explained by higher levels of circulating mAb. Indeed, dose escalation based on the level of skin toxicity showed a possible avenue for improved efficacy [9].

We hypothesize that response to treatment is dependent on uptake of cetuximab in tumor lesions. Differences in biodistribution and tumor uptake of the antibody can be evaluated by immunoPET imaging as demonstrated by successful proof-of-principle studies in humans [10, 11]. The half life of the radiotracer ^89^Zr (t_1/2_ = 78.4 h) matches the biological half-life of intact antibodies with slow kinetics like cetuximab. In a preclinical study with tumor-bearing mice, ^89^Zr-cetuximab uptake was demonstrated in EGFR-positive tumors. ^89^Zr-cetuximab uptake did not correlate with EGFR expression levels, implying that pharmacokinetic and pharmacodynamic factors might influence cetuximab accumulation in the tumor [12].

We performed ^89^Zr-cetuximab PET imaging in patients with wt K-RAS mCRC with an indication for anti-EGFR mAb monotherapy to investigate biodistribution and tumor uptake as well as to establish the optimal scanning time point to visualize tumor targeting. Most importantly, we evaluated whether uptake on ^89^Zr-cetuximab PET imaging can discriminate between patients responding to treatment with cetuximab versus non-responding patients.

## RESULTS

Ten patients with wt K-RAS mCRC and an indication for cetuximab monotherapy were enrolled. A table with patient characteristics is available online (S2). No ^89^Zr-cetuximab related toxicity was reported. Only known adverse events to cetuximab were observed, such as skin toxicity, hypomagnesaemia and infusion related reactions, none exceeding grade 2.

**Figure 1:**
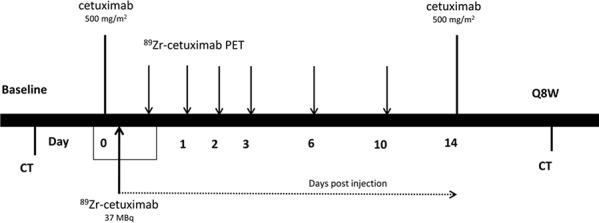
Timeline

**Figure 2:**
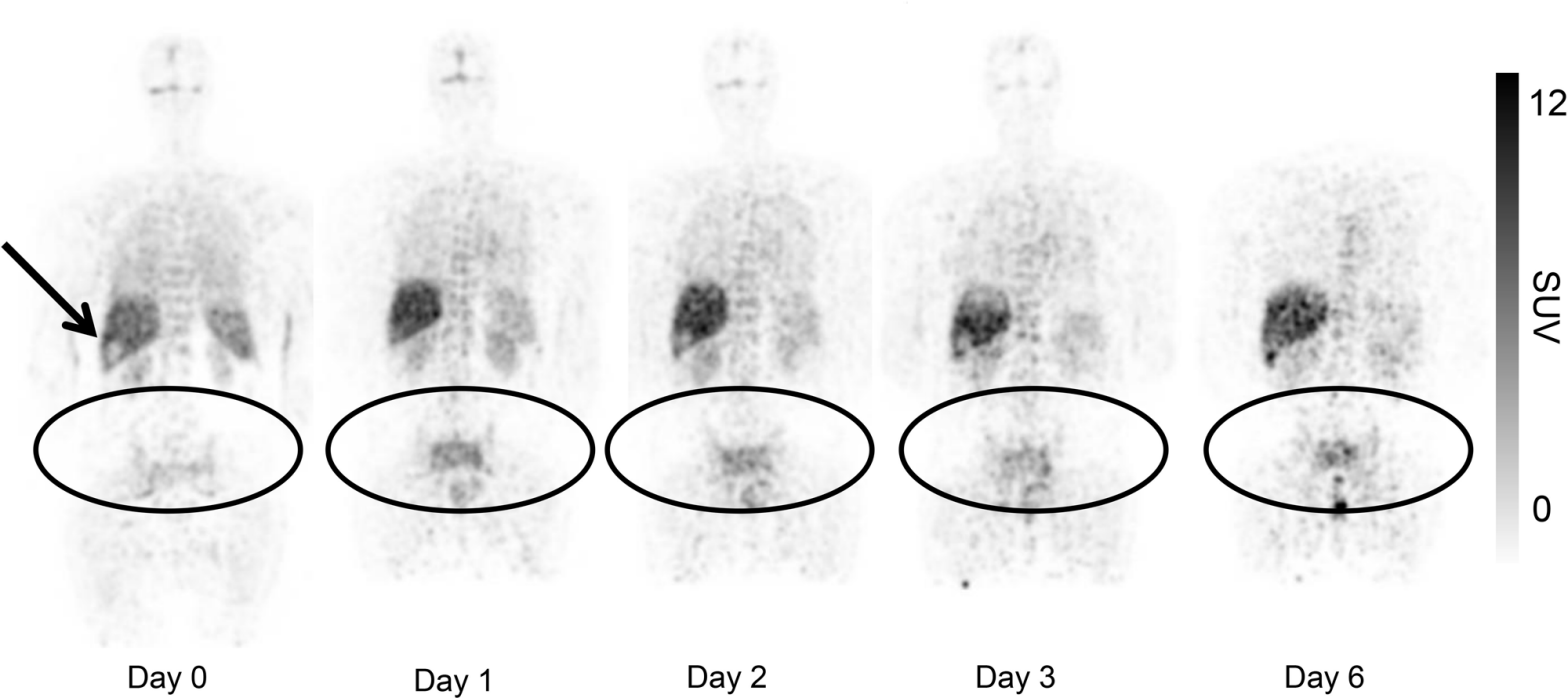
Uptake of ^89^Zr-cetuximab in patient 3 with tumor lesions in the pelvis and sacral bone Presented images are with equal SUV max (decay corrected). Visual inspection shows uptake in normal organs which is decreasing over time. ^89^Zr-cetuximab is sequestered in liver, a relatively photopenic lesion is observed at the site of a liver metastasis (arrow). Accumulation of ^89^Zr-cetuximab over time is demonstrated in the tumor lesions. On the last scan a rectal hotspot with excreted ^89^Zr in feces is seen. Due to positioning of the patient in the scanner the head and neck region is not visible in this plane.

**Figure 3:**
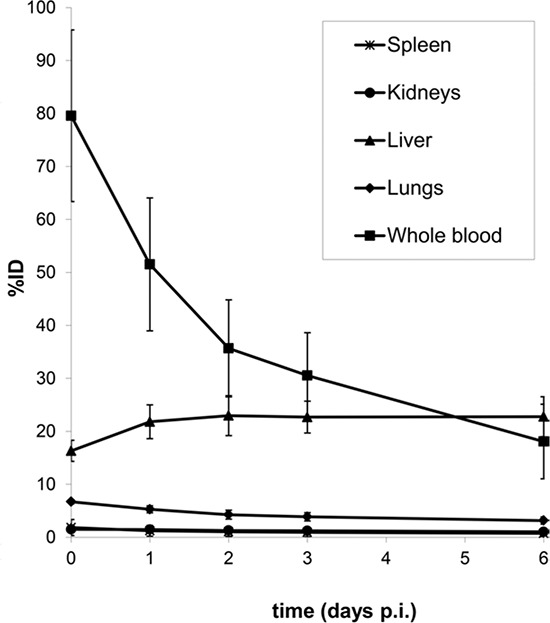
Biodistribution (%ID) of ^89^Zr-cetuximab as a function of time (days p.i.) for kidney, liver, lung, spleen and whole blood Data are image derived and decay corrected. Error bars denote the standard deviation. (*n* = 7)

Whole body (WB) images, acquired at consecutive time points after administration of ^89^Zr-cetuximab (Figure 1 Timeline), showed radioactivity in blood pool, liver, kidney, spleen, intestine and bone marrow. We observed no visible uptake in the skin (Figure 2). The % injected dose (ID) (decay corrected) in spleen, kidneys and lungs as well as blood pool decreased in time. In liver, the %ID increased during the first two days, after which uptake plateaued at approximately 23% of ID (SD 4%) (Figure 3), with a marked increase in organ to blood pool ratio. Radioactivity concentration as measured in the blood samples correlated well with the image derived input (R^2^ = 0.97; S3). At day 6 p.i. the total radioactivity retrieved from the WB PET images had decreased by 18.5% compared to the first scan due to gastrointestinal excretion, as no excretion via the bladder was observed.

In 6 out of 10 patients, target lesions were visually assessed positive for ^89^Zr-cetuximab uptake. Figure 4A and 4B shows examples of visible ^89^Zr uptake in a metastatic lesion of the iliac bone (patient 8) and the lung (patient 10). In Figure 4C, another lung lesion in patient 10 shows no uptake. Most tumor lesions showed increasing uptake in time, indicating accumulation of cetuximab. SUV_peak_ of these lesions varied between 2.2–7.5 on day 6 p.i. Figure 4D illustrates the photopenic aspect of liver metastases within normal liver tissue accumulating high amounts of ^89^Zr-cetuximab. Two of the 3 patients who were scanned at day 10 p.i. had visible ^89^Zr-cetuximab uptake. SUV_peak_ at day 10 increased compared to day 6 in patient 8 (from 7.3 to 10.3), but was comparable in patient 6 (3.17 and 3.36, Figure 4E Due to the physical half-life of ^89^Zr, image quality deteriorated over time, making day 6 p.i. the optimal scanning time point. Visually negative tumor sites had SUV_mean_ of 1.0–1.9 at day 6 p.i. (Figure 5).

**Figure 4A:**
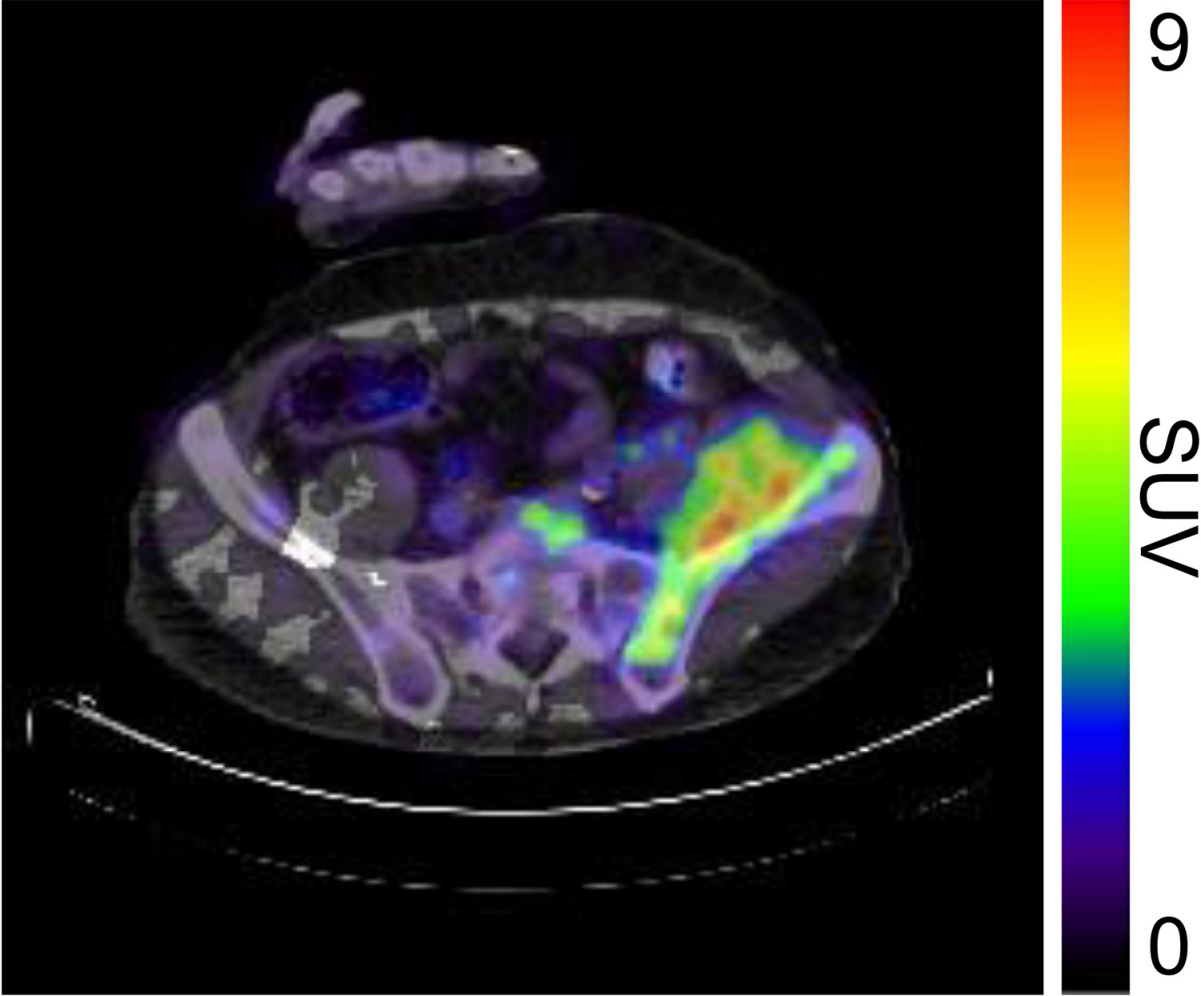
^89^Zr-cetuximab PET scan of patient 8 at day 6 p.i. with visible uptake in tumor lesion in the left iliac bone

**Figure 4B:**
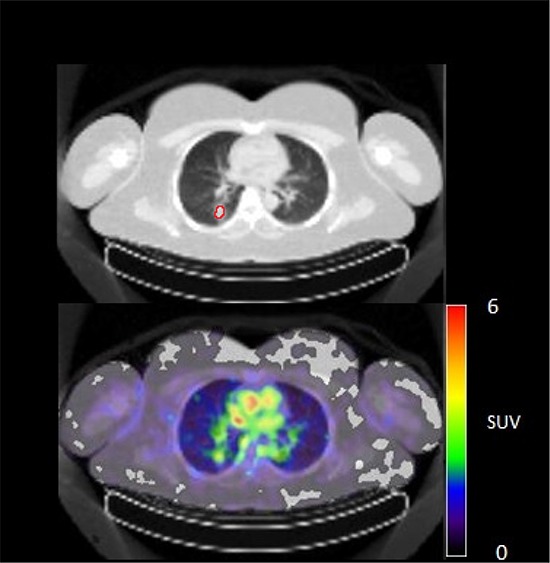
^89^Zr-cetuximab PET scan of patient 10 at day 6 p.i. with visible uptake in tumor lesion in the lower lobe of the right lung and low accumulation in surrounding healthy lung tissue

**Figure 4C:**
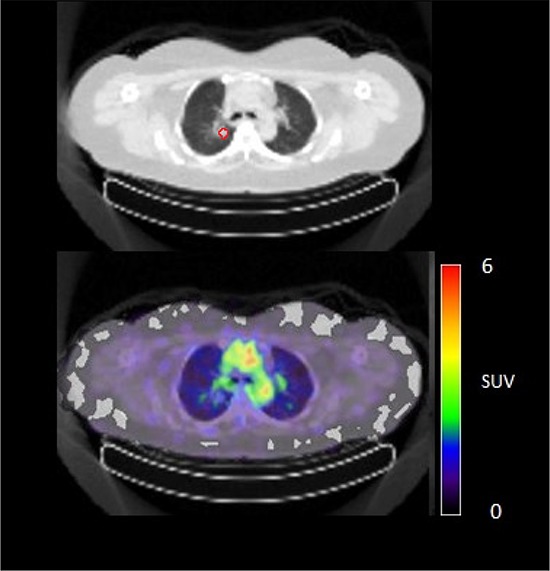
^89^Zr-cetuximab PET scan of patient 10 at day 6 p.i. without visible uptake in tumor lesion in the upper lobe of the right lung

**Figure 4D:**
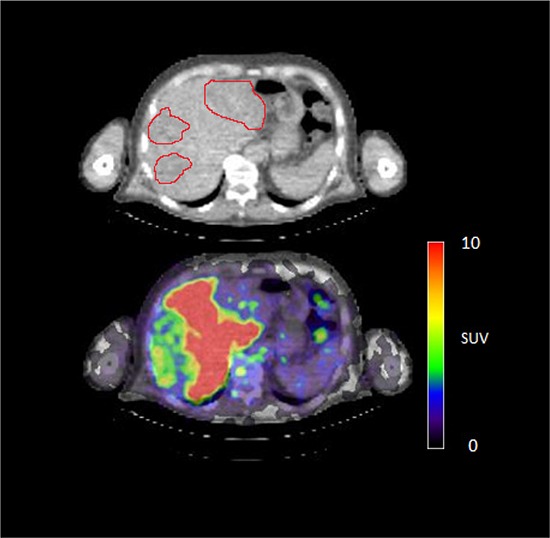
^89^Zr-cetuximab PET scan of patient 3 at day 6 p.i. illustrating high accumulation in healthy liver with relative photopenic area's in metastases

**Figure 4E:**
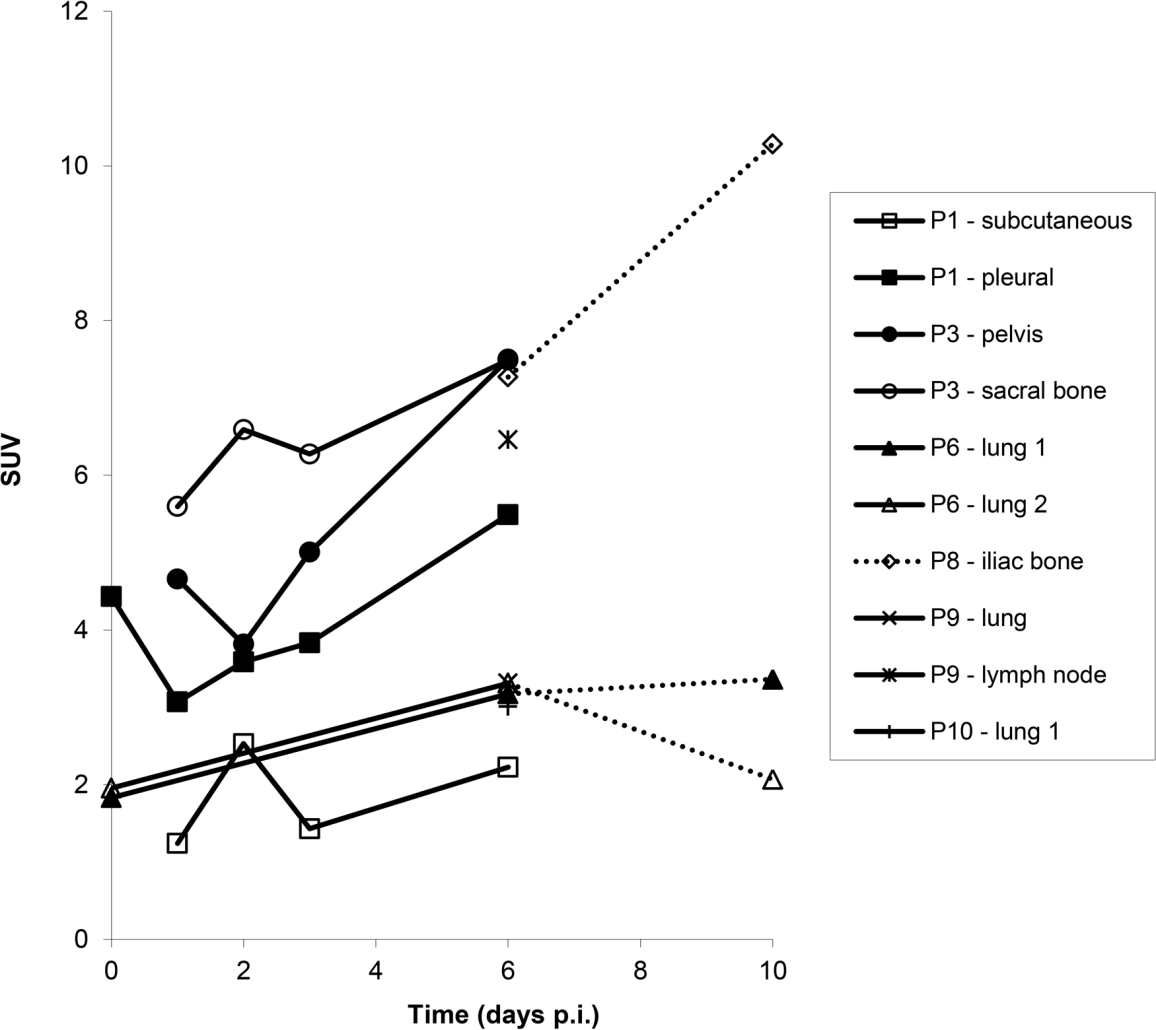
SUV_peak_ calculated for tumor lesions with visible ^89^Zr-cetuximab uptake at sequential scanning time points

**Figure 5:**
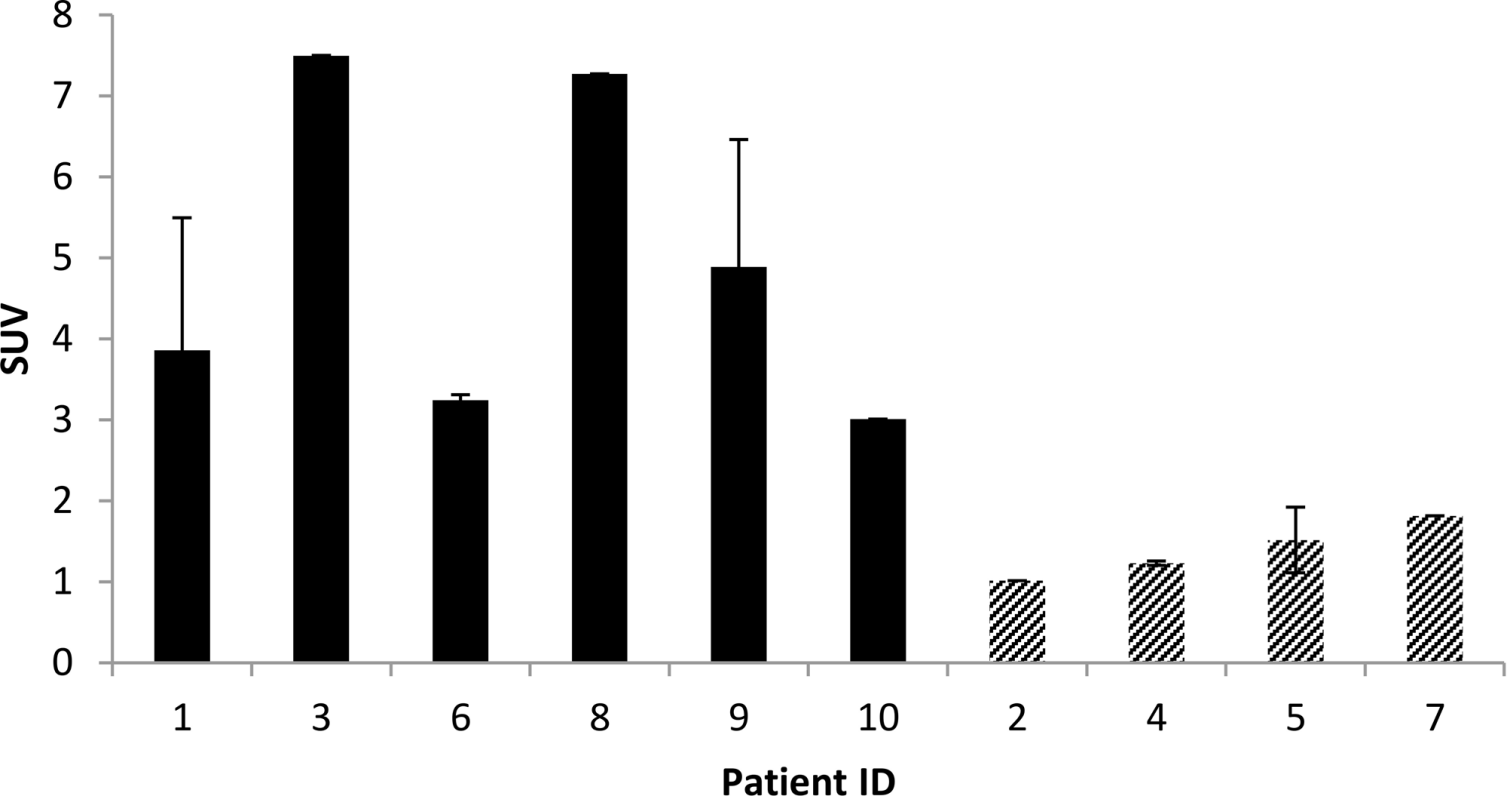
Average SUV_peak_ of target lesions on day 6 p.i. Filled bars represent patients with visible ^89^Zr-cetuximab uptake, dashed bars represent lesions with no visible uptake Patient ID based on chronological order of inclusion.

The majority of patients had 2 evaluable lesions and in all but one patient, ^89^Zr-cetuximab tumor uptake was either present or absent in both lesions. Five patients had stable disease according to RECIST 1.1. Of 6 patients with visible tumor uptake of ^89^Zr-cetuximab, 4 experienced meaningful clinical benefit. Three of 4 patients without visible uptake had progressive disease at first evaluation at 8 weeks after start of treatment (Table 1).

**Table 1:** ^89^Zr-cetuximab uptake in extrahepatic target lesions

Patient	Extrahepatic target lesions	^89^Zr uptake	Clinical benefit
1	Pleura	+	−
	Subcutaneous	+	
2	Lymphnode	−	−
	Lung	−	
3	Pelvic bone	+	+
	Sacral bone	+	
4	Adrenal gland	−	+
	Soft tissue	−	
5	Adrenal gland	−	−
	Lymph node	−	
6	Lung (1)	+	−
	Lung (2)	+	
7	Primary tumor	−	−
8	Iliac bone	+	+
9	Lymph node	+	+
	Lung	+	
10	Lung	+	+
	lung	−	

## DISCUSSION

We evaluated ^89^Zr-cetuximab PET imaging in patients with wt K-RAS mCRC and found tumor uptake of ^89^Zr-cetuximab in 6 out of 10 patients of whom 4 had clinical benefit of cetuximab treatment (Table 1). Based on the design of this clinical trial in which we expected uptake in ≥1 of 10 or ≤7 of 10 patients (power >90%, type I error <5%), our results indicate that tumor uptake of ^89^Zr-cetuximab may be used to predict clinical benefit of cetuximab in patients with wt K-RAS mCRC, which should be further validated in a larger cohort of patients.

Previously, a dosimetry study of ^99m^Tc-C225 (= cetuximab) in patients with squamous cell carcinoma of the head and neck showed reasonable dosimetric properties, however, tumor uptake was not evaluated in this trial [13].

In order to optimally reflect the biodistribution of the mAb in patients, immediate binding of the labeled mAb to easy accessible non tumor sites, e.g. liver, should be minimized. A study evaluating ^111^In-C225 in patients with squamous cell lung carcinoma showed that liver sequestration of ^111^In -C225 decreased from 32 to 21.6 %ID with increasing dose of unlabeled C225 (up to 300 mg). Furthermore, increasing doses of unlabeled C225 resulted in higher tumor uptake of ^111^In-C225 [14]. Similar results were obtained with ^89^Zr-trastuzumab directed against HER2. In trastuzumab-naive patients administration of only 10 mg unlabeled trastuzumab resulted in a relatively high uptake in the liver, whereas imaging characteristics were optimal when 50 mg unlabeled trastuzumab was administered [11].

As a proof of principle, we administered a scouting dose of 0,1 mg ^89^Zr-cetuximab before the unlabeled therapeutic dose of cetuximab in three patients. In blood samples taken 2 and 3 hours after administration of the scouting dose, only <10 %ID ^89^Zr-cetuximab could be detected. However, by administration of the therapeutic dose of 500 mg/m^2^ cetuximab before the labeled fraction, sufficient ^89^Zr-cetuximab was found in the blood pool for tumor targeting (80% ID, see Figure 2). In addition, the half life of ^89^Zr-cetuximab if co-administered with the therapeutic dose, is comparable to unlabeled cetuximab, indicating that in our model ^89^Zr-cetuximab reflects biodistribution of unlabeled cetuximab.

Tumor uptake was initially evaluated by visual assessment, which implies contrast with background activity. The optimal scanning time point appears to be day 6 p.i., which is in line with literature and our expectations, based on the t½ of ^89^Zr [10]. The subsequently calculated SUV_peak_ at day 6 p.i. can discriminate between lesions with and without visible uptake (Figure 5) and suggests that a cut-off SUV_peak_ could be helpful in the determination of specific uptake versus background activity. Because a significant amount of the ID of ^89^Zr-cetuximab accumulated in the liver, hepatic metastases – although large enough for imaging purposes (diameter 4–14cm) - were unsuitable to evaluate tumor uptake as spill-over from uptake in adjacent normal liver tissue hampered adequate uptake evaluation of tumor sites. In addition, many large lesions have central necrosis with only a rim of viable tumor tissue, which is located immediately adjacent to healthy liver tissue accumulating very high levels of ^89^Zr cetuximab. As liver is a common metastatic site of mCRC, we have attempted to quantify uptake in hepatic metastases. In 6 target lesions in 5 patients we observed transient accumulation of ^89^Zr-cetuximab with highest levels at day 2 pi showing a comparable pattern as healthy liver tissue (data not shown). As the uptake pattern largely followed normal liver tissue, quantification of hepatic lesions seems to be unreliable due to spill-over of adjacent liver tissue. With the liver being a common metastatic site of mCRC this can limit the use of ^89^Zr-cetuximab as a treatment selection tool.

The data in this study are too limited to draw conclusions on the correlation between blood concentration, liver uptake, tumor targeting and response. However, three patients who did not show uptake had progressive disease at first response evaluation. One could postulate that insufficient cetuximab was available for uptake in tumor lesions due to sequestration in the liver or other EGFR expressing organs. For example, one patient who had no visible ^89^Zr-cetuximab uptake in target tumor lesions, had rather high liver uptake (29.0 %ID, average all patients 22.8 ± 3.5 %ID) and relatively low plasma levels at day 6 p.i. (10.0 %ID, average all patients 18.1 ± 6.5 %ID) suggesting possible inadequate availability in tumor tissue.

Of 6 patients showing ^89^Zr-cetuximab uptake, 4 had clinical benefit. When comparing patient 6, 9 and 10 who all had lung metastases showing uptake, only patient 9 and 10 had clinical benefit. The lack thereof for patient 6 may be due to the multiple lines of previous therapy including radiotherapy on the lung metastases compared to 1–2 previous lines of therapy for the other two patients leading to a potential difference in tissue architecture and cellular content of these lesions. The absence of response may also be due to N-RAS or other mutations, however, unfortunately no adequate tumor material was available for further assessment of the mutational status in these patients. One patient had clinical benefit, although ^89^Zr-cetuximab uptake could not be visualized. Possibly, the amount of cetuximab that reached the tumor was insufficient for visual assessment, but did induce anti-tumor activity, for example by antibody-dependent cell-mediated cytotoxicity [15]. For 7 patients with two lesions available for quantification only patient 10 showed heterogeneous uptake of ^89^Zr-cetuximab (Figure 4B and 4C). Although all lesions in this patient showed response to treatment, one of the lung lesion did not show visible uptake of ^89^Zr cetuximab. This might be caused by a difference in size, the negative lesion is smaller compared to the others and thereby relatively unfavorable for ^89^Zr PET imaging.

In conclusion, PET-imaging with ^89^Zr-cetuximab is feasible. Despite relatively high liver uptake, variable tumor uptake can be demonstrated in extra-hepatic metastases of patients with wt K-RAS colorectal carcinoma by visual assessment of ^89^Zr-cetuximab PET scans. The optimal scanning time point appears to be at day 6 post radiotracer injection. With 6 of 10 patients showing uptake, statistical conditions were met for ^89^Zr-cetuximab imaging to qualify as a potential treatment selection tool, however additional data are needed to confirm this

We are currently investigating whether tumor uptake on ^89^Zr-cetuximab PET scan could guide dose escalation to improve the clinical response (NCT02117466). Ultimately, we aim to develop a PET-imaging guided tool to select patients who could benefit from cetuximab treatment.

## MATERIALs AND METHODS

### Patients

Patients with histologically proven exon 2 K-RAS wt mCRC, were eligible if they had progressive disease after standard first and second line treatment (fluoropyrimidines, oxaliplatin and irinotecan) or had contra-indications to these agents. Only K-RAS exon 2 mutations were tested prior to inclusion because the trial was started prior to the publication on the importance of other K-RAS or N-RAS mutations [6]. Eligible patients had ECOG of 0–2, a life expectancy of at least 12 weeks, good end-organ function, and one or more measureable lesion outside the liver according to RECIST 1.1. Prior anti-EGFR therapy as well as skin conditions interfering with EGFR inhibition were exclusion criteria amongst others. The study (NCT01691391) was reviewed and approved by the Central Committee on Research Involving Human Subjects of the Netherlands and the Medical Research Ethics Committee of the VU University Medical Center, the Netherlands. All patients gave written informed consent prior to any study specific procedures.

### ^89^Zr-cetuximab

^89^Zr has been produced and purified as described before and is coupled to mAbs via the bifunctional chelate desferal (Df), [16, 17] which has been safely used in the clinic before [10]. ^89^Zr-cetuximab is produced in compliance with current Good Manufacturing Practice at the VU University Medical Center. The procedures for radiolabeling of cetuximab with ^89^Zr have been validated with respect to the final quality of the prepared conjugate. Details can be found in supplementary data online (S1).

### Treatment with cetuximab

Patients were treated with 500 mg/m^2^ cetuximab administered intravenously every two weeks. Adverse events were graded according to CTCAE v4. Tumor response was analyzed every 8 weeks according to RECIST 1.1 (Figure 1). Treatment was ended in case of unacceptable adverse events, worsening symptoms of disease, clinical or radiological disease progression, request by the patient or death.

### ^89^Zr-cetuximab PET

Within 2 hours after the first administration of 500 mg/m^2^ unlabeled cetuximab, 10 mg of ^89^Zr-cetuximab (37 ± 1 MBq) was injected. The injected dose ^89^Zr (MBq) was corrected for residual activity in the syringe and needle. Whole-body (WB) PET scans (mid-femur-skull vertex) were acquired 1–2 hours and 1, 2, 3 and 6 days post injection (p.i.) in 7 patients and 6 and 10 days p.i. in 3 patients (Figure 1). At every scanning time point, venous blood samples were taken for pharmacokinetic purposes. A 35 mAs low-dose (LD) CT scan was acquired for attenuation correction and localization purposes. PET scans consisted of 10–12 bed positions, of 5 min each. PET data were corrected for dead time, scatter, randoms, decay, and tissue attenuation and reconstructed according to Makris et al [18].

An [^18^F]-FDG PET/CT was performed at baseline to identify target lesions. ^89^Zr-cetuximab PET images were visually assessed for ^89^Zr-cetuximab uptake in target lesions. Images were evaluated by a nuclear medicine physician (OSH) and a medical oncologist (CWM). During the first reading session the nuclear medicine physician was blinded for clinical information on target lesion distribution. Tumors were scored as either positive or negative for ^89^Zr-cetuximab uptake by consensus, as a function of tracer uptake versus direct background.

### Quantification of uptake

For quantification of radiotracer accumulation in organs, regions of interest (ROI) were drawn manually on the ^89^Zr-cetuximab PET images or the co-registered LD CT scan if organ delineation was unclear on PET. Average activity concentration (AC) was measured and percentage of injected dose (%ID) was calculated. Image derived AC in the blood pool was calculated from fixed-size ROI (total volume ∼1.6 mL) placed in the middle of the aortic arch on LD CT, on 5 consecutive planes. AC was measured and %ID was calculated based on estimated blood volume.

Radiotracer accumulation in tumors was calculated by drawing ROIs on the PET images and Standardized Uptake Value (SUV) corrected for body weight was calculated from the measured AC. In tumors with visible uptake, SUV_peak_ was calculated [18, 19]. For tumor lesions without visible uptake, an average background activity was measured in a 5 cm diameter ROI in the area of the tumor lesion and SUV_mean_ was calculated. WB ROIs were drawn to calculate the total activity measured in the acquired PET images. Total activity measured on day 6 p.i. was compared to the activity at 1 hr p.i. to evaluate excretion of ^89^Zr-cetuximab. AC was corrected for decay between the time of injection and the start time of the scan.

### Statistics

Based on the clinical benefit rate of single agent therapy with cetuximab in wt K-RAS patients and the assumption that uptake is related to response, we hypothesized that 40% of patients would show ^89^Zr-cetuximab uptake in tumor lesions [3–5]. If this is correct, we expected uptake in ≥1 of 10 or ≤7 of 10 patients (power >90%, type I error <5%).

## SUPPLEMENTARY DATA


