# Supplementary material for: 89Zr-cetuximab PET imaging in patients with advanced colorectal cancer
Source: Oncotarget. 2015 Jul 23;6(30):30384–93. doi: 10.18632/oncotarget.4672 (PMC4745807; doi:10.18632/oncotarget.4672)
Supplement: Supplementary file 1 [file oncotarget-06-30384-s001.pdf]

SUPPLEMENTARY DATA

^{89}ZR-CETUXIMAB LABELLING PROCEDURE

^{89}Zr has been produced and purified as described before and is coupled to mAbs via the bifunctional chelate desferal (Df), [13] which has been safely used in the clinic before.[10] In the conjugation of cetuximab with desferal 3 equivalents of chelator have been used. On average 1.5 desferal groups are coupled per antibody molecule as determined by SEC-HPLC at 430 nm. In the radiolabeling ~ 2.5 mg DFO-cetuximab is used. After radiolabeling and taking the right amount of Zr-89 this is brought up to 10 mg with cold cetuximab.

^{89}Zr-cetuximab is produced in compliance to current Good Manufacturing Practice at the VU University Medical Center. The procedures for radiolabeling of cetuximab with ^{89}Zr have been validated with respect to the final quality of the prepared conjugate. The mean labeling efficiency was 85.4 ± 3.7%. The mean percentage of label bound to the antibody as assessed by TLC was 97.9 ± 0.9%, while the mean immunoreactive fraction as assessed by cell binding assay[16] was 95.4 ± 3%. Sterility of each ^{89}Zr-cetuximab batch was assured by performing a media fill immediately after final filter sterilization of each batch. These procedures resulted in a sterile final product with endotoxin levels < 2.5 EU/mL.

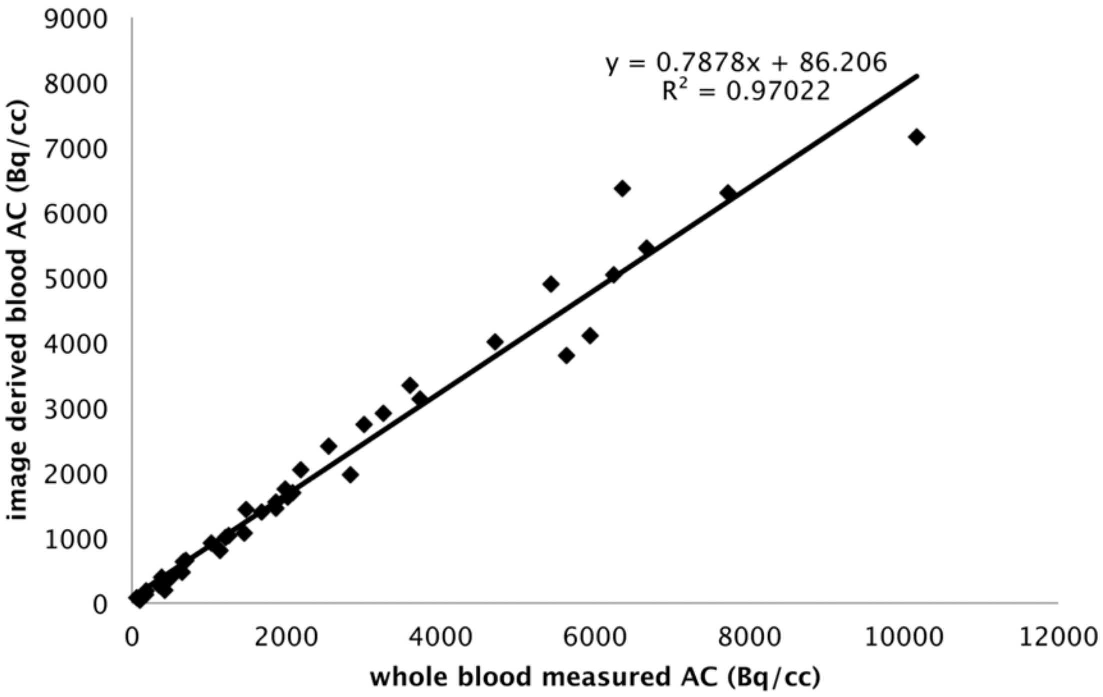

Supplementary Figure S1: Correlation of whole blood measured and image derived blood Activity Concentrations (AC) in Bq/ml.

Supplementary Table S1: Patient characteristics

patient	sex	age	primary tumor	metastases	prior therapy		
					adjuvant	palliative	
						1st line	2nd line
1	F	66	colon	pleura, lung, subcutaneous	folfox	irinotecan	
2	M	73	sigmoid	lymph nodes, liver, lung		capox-b	irinotecan
3	M	54	rectum	lymph nodes, liver, sacral bone, pelvic bone		capox- b	folfiri
4	M	72	sigmoid, local recurrence	liver, adrenal gland, tumor deposit		capox-b	irinotecan
5	M	61	coecum	lymph nodes, adrenal gland, peritoneal deposit		folfox	irinotecan
6	F	66	rectum	lymph nodes, lung	capox	capox-b	capiri
7	M	52	sigmoid	lymph nodes, liver		capox-b	folfiri
8	M	58	rectum	iliac bone		capox	irinotecan
9	F	50	rectum	liver, lung, lymphnodes		capox-b	
10	F	54	sigmoid	lung, liver		folfox-b	irinotecan

capox: capecitabin and oxaliplatin, capox-b: capox and bevacizumab, folfox: 5FU/LV and oxapliplatin, folfiri: 5FU/LV and irinotecan, capiri: capecitabin and irinotecan
